# Validation and factor structure of the Japanese version of the inventory to diagnose depression, lifetime version for pregnant women

**DOI:** 10.1371/journal.pone.0234240

**Published:** 2020-06-11

**Authors:** Chika Kubota, Toshiya Inada, Yukako Nakamura, Tomoko Shiino, Masahiko Ando, Branko Aleksic, Aya Yamauchi, Mako Morikawa, Takashi Okada, Masako Ohara, Maya Sato, Satomi Murase, Setsuko Goto, Atsuko Kanai, Norio Ozaki

**Affiliations:** 1 National Center of Neurology and Psychiatry, Kodaira, Tokyo, Japan; 2 Department of Psychiatry, Nagoya University Graduate School of Medicine, Nagoya, Aichi, Japan; 3 Department of Psychiatry and Psychobiology, Nagoya University Graduate School of Medicine, Nagoya, Aichi, Japan; 4 Division of Developmental Emotional Intelligence Research Center for Child Mental Development, University of Fukui, Fukui, Japan; 5 Center for Advanced Medicine and Clinical Research, Nagoya University Graduate School of Medicine, Nagoya, Aichi, Japan; 6 Liaison Medical Marunouchi, Nagoya, Aichi, Japan; 7 Goto Setsuko Ladies Clinic, Nagoya, Aichi, Japan; 8 Graduate School of Education and Human Development, Nagoya University, Nagoya, Aichi, Japan; Chiba Daigaku, JAPAN

## Abstract

**Introduction:**

A history of major depressive disorder before pregnancy is one risk factor for peripartum depression. Therefore, the purpose of the present study was to examine the validation and factor structure of the Japanese version of the Inventory to Diagnose Depression, Lifetime version (IDDL) for pregnant women.

**Methods:**

The study participants were 556 pregnant women. Factor analysis was performed to identify the factor structure, construct validity was examined based on the results of the factor analysis, and reliability was examined using Cronbach’s α coefficient.

**Results:**

Based on the results of the factor analysis of the IDDL, a bifactor model composed of a single general dimension along with the following five factors was extracted: (1) depression, anxiety, and irritability (items 1, 2, 8–10, and 19–21); (2) retardation, decreased concentration, indecisiveness, and insomnia (items 4, 11, 12, and 17); (3) decrease in appetite/significant weight loss (items 13 and 14); (4) increase in appetite/significant weight gain (items 15 and 16); and (5) diminished interest, pleasure, and libido (items 5–7). Cronbach’s α coefficients for these five factors were as follows: 0.910, 0.815, 0.780, 0.683, and 0.803, respectively.

**Conclusions:**

The reliability, construct validity, and factor structure of the Japanese version of the IDDL were confirmed in pregnant women.

## Introduction

Mental health care in the perinatal period is important. A multicenter prospective study estimated that the prevalence of perinatal depression in Japan was 5.6% during pregnancy and 5.0% during the postnatal period [1]. In 2017, it was reported that suicide is the most frequent cause of maternal death in Tokyo, Japan [2]. The existence of perinatal depression has been pointed out with this background. Hence, the prevention of perinatal depression is urgently needed. Therefore, preventive activities such as screening in maternal health examinations and home visits by public health nurses are being promoted for the early detection of and intervention for perinatal depression.

A meta-analysis reported that a history of major depressive disorder (MDD) is a major risk factor for perinatal depression [3, 4]. Confirming the history of previous depressive episodes is considered to be useful in the prevention of perinatal depression; however, currently, this is not sufficiently performed in primary care in Japan, such as in maternal health examinations.

The Inventory to Diagnose Depression, Lifetime version (IDDL) is a self-administered questionnaire developed by Zimmerman et al. in 1987 to assess lifetime history of MDD based on the Diagnostic and Statistical Manual of Mental Disorders (DSM)-III [5]. The diagnostic interviews usually require sufficient rater training for stable and reliable evaluations, and a limited number of subjects who can be evaluated within the given time. Therefore, self-administered questionnaires are more useful than diagnostic interviews for medical examinations.

Although both the original English and the Japanese version of the IDDL have been validated [5, 6], reports examining the reliability and validity of the IDDL are quite limited and have had relatively small sample sizes. Moreover, the factor structure of the IDDL has scarcely been studied. Since August 2004, we have been conducting a prospective cohort study in Nagoya involving women from pregnancy to 1 month postpartum. In the present study, using these data, the validity and factor structure of the Japanese version of the IDDL were examined using a relatively large sample of pregnant women. The symptomatologic features of depression were also discussed based on the results of the obtained factor structure.

## Methods

### Study design

In the present study, which started in August 2004 in Nagoya, cross-sectional data on perinatal depression were extracted from the prospective cohort. In this prospective cohort study, a three-factor structure of depression, anxiety, and anhedonia was revealed based on the Edinburgh Postnatal Depression Scale (EPDS) [7].

### Participants

The IDDL has been used at four hospitals in Nagoya—one general hospital (Nagoya Teishin Hospital), two obstetrics and gynecology hospitals (Kaseki Hospital and Royal Bell Clinic), and one university hospital (Nagoya University Hospital)—since May 2011. The eligibility criteria were: pregnant female aged 20 years or older, ability to read and write Japanese, and attended a gynecological checkup at one of the four hospitals mentioned above.

### Ethical considerations

The study protocol was approved by the ethics committee of Nagoya University Hospital. All study procedures were conducted in accordance with the Declaration of Helsinki and other relevant ethical guidelines, and written informed consent was obtained from all participants.

### Measurements

The following demographic and socioeconomic data were collected: age, years of education, number of births, economic status, and employment status. All participants were assessed using self-administered questionnaires (the Japanese versions of the EPDS and IDDL).

### Edinburgh Postnatal Depression Scale (EPDS)

The EPDS is a self-administered questionnaire designed by Cox et al. in 1987 [8] to screen for postpartum depression. It is composed of 10 items scored on a 4-point Likert scale, with scores ranging from 0–3. The Japanese version of the EPDS was translated from its original English version, and validated by Okano et al. in 1996 [9]. During pregnancy, the sensitivity, specificity, and positive predictive value of the EPDS with a cutoff point of 12/13 were 90%, 92%, and 55%, respectively [10]. The Japanese version of the EPDS has been shown to have a stable factor structure (depression [items 7 and 9], anxiety [items 4 and 5], and anhedonia [items 1 and 2]) from pregnancy to the postpartum period [7].

### Inventory to Diagnose Depression, Lifetime version (IDDL)

The IDDL is a self-administered questionnaire developed by Zimmerman et al. to assess lifetime history of MDD in accordance with the DSM-III [5]. It is composed of 22 symptom items scored on a 5-point Likert scale, with scores ranging from 0–4. When the score of an individual symptom item was 1 or higher, the participants was asked whether the symptom lasted longer than 2 weeks.

The following evaluation method was used in the adopted Japanese version of the IDDL, the same as that used in the original English version. First, if a symptom continues for more than 2 weeks, the respondent is considered as having that symptom. Second, a score of 2–4 indicates the presence of a specific symptom for each item, except items 5 and 6, for which a score of 3–4 is used. Third, all 22 items on the IDDL are classified into nine depressive symptoms as defined by the DSM-III. The two major symptoms of four items are: (1) depressive mood (items 1 and 20) and (2) diminished interest or pleasure (items 5 and 6). The remaining seven depressive symptoms are: (3) decrease or increase in appetite/significant weight loss or weight gain (items 13–16), (4) insomnia or hypersomnia (items 17 and 18), (5) psychomotor agitation or retardation (items 3 and 4), (6) fatigue or loss of energy (items 2 and 7), (7) feelings of worthlessness or excessive inappropriate guilt (items 8 and 9), (8) diminished ability to think or concentrate, or indecisiveness (items 11 and 12), and (9) recurrent suicidal thoughts (item 10). In children and adolescents, item 21 is used in place of item 20. Items 19 and 22 are not applied for the DSM-III diagnosis. Whether symptoms have continued for 2 weeks is checked to diagnose MDD as defined by the DSM-III for each item. Finally, the conditions regarding a history of MDD were as follows: for items that last 2 weeks or more, (1) out of 22 items, five or more have the cutoff score or higher, and (2) these five or more items contain one or more items of the two major symptoms.

The original English version of the IDDL was validated by Zimmerman et al. as follows [5]. For 164 participants, concurrent validity was examined using the Diagnostic Interview Schedule (DIS) [11], and reliability was evaluated using Cronbach’s α and Spearman–Brown coefficients. As a result, it was confirmed to have good internal consistency (α = 0.92) and split-half reliability (Spearman–Brown coefficient = 0.90). The lifetime prevalence of MDD was not significantly higher in the IDDL than in the DIS, and the sensitivity and specificity were 74% and 93%, respectively (kappa between the IDDL and DIS = 0.60).

In Japan, Uehara et al. translated the original English version into Japanese. It was confirmed through a back-translation process and validated as follows [6]. Thirty normal participants, 30 participants with a history of MDD, and 29 with a history of anxiety disorders diagnosed using the Structured Clinical Interview for DSM-III-R (SCID) [12] were enrolled as participants. Regarding reliability, internal consistency as evaluated using Cronbach’s α was 0.86, and split-half reliability as evaluated by the Spearman–Brown coefficient was 0.76. Concurrent validity was examined using the SCID. For the 30 patients with a history of MDD and the 30 normal participants, the sensitivity and specificity were 83% and 97%, respectively. For the 30 patients with a history of MDD and the 29 with a history of anxiety disorders, the sensitivity and specificity were 83% and 60%, respectively.

However, the factor structure of the IDDL for both the original English and the Japanese version remains unclear.

### Statistical analysis

The rate of missing data for all items was calculated, and Little’s missing completely at random (MCAR) test was performed to decide how to deal with the missing data [13].

To examine construct validity, a factor analysis was conducted after a measure of specimen validity using the Kaiser–Meyer–Olkin (KMO) Test and Bartlett’s test of sphericity [14]. A KMO value above 0.6 and a value below 0.05 on Bartlett’s test of sphericity are appropriate for conducting a factor analysis. The participants were randomly divided into two sample sets. The first was used for exploratory factor analysis (EFA), and the second for confirmatory factor analysis (CFA). For the EFA, a screen test was used to determine the number of factors, and maximum likelihood estimation with promax rotation was performed. EFA was conducted for the items for which the communalities were over 0.4 [15]. The items of the IDDL with a factor coefficient level above 0.30 were defined as being included in the same factor. If there were items with a coefficient level more than 0.30 in multiple factors, that item was included as a factor of a higher coefficient level. CFA was carried out based on the models obtained by EFA, and the best-fit model was determined among the six models, including the 1-, 2-, 3-, 4-, and 5-factor models and the bifactor model. The following fit indices were used to assess model fit: chi-square (*χ*^2^)/degrees of freedom (*df*), comparative fit index (CFI) [16], root mean square error of approximation (RMSEA) [17], and Akaike’s information criterion (AIC) [18]. A CFI value above 0.97 is considered good, and above 0.95 acceptable; 0.90 or more is required for the adoption of the model [16]. An RMSEA value below 0.05 is considered good, and below 0.08 acceptable; less than 0.1 is required for the adoption of the model [17]. Lower *χ*^2^/*df* and AIC values are considered to indicate a better fit [18]. Whether the goodness of fit was sufficiently improved by increasing the number of factors was evaluated based on the Δ*χ*^2^ value, using the table of *χ*^2^ distribution.

To examine internal reliability, after the best-fit factor structure had been clarified, the Cronbach’s α coefficients of individual factors were calculated. Cronbach’s α coefficients above 0.7 are acceptable [19].

To clarify how a history of MDD affects depression during pregnancy, based on the method by Zimmerman et al. [5, 6], pregnant women with a history of MDD were extracted using the IDDL. Then, the pregnant women with a cutoff score of 13 or more on the EPDS, as proposed by Usuda et al. [10], were identified.

The data were analyzed using SPSS Statistics version 26.0 and SPSS Amos version 26.0 (IBM Tokyo, Japan).

## Results

### Characteristics of the participants

Little’s MCAR test indicated that the data were not randomly missing (p < 0.001); therefore, the multiple imputation method was used to handle missing data in the demographic analysis. Two participants were excluded because more than half of the IDDL items were missing. Multiple imputation was used to create and analyze 10 multiply imputed data sets.

As a result, 556 of 558 women were analyzed in this study (mean [M]: 22.6 weeks gestation, standard deviation [SD]: 6.1 weeks). The mean age of the participants was 32.9 (SD: 4.8) years, and that of their partners was 35.1 (SD: 5.8) years. The mean years of education was 15.0 (SD: 1.9). The ratios of nulliparas, primiparas, and those who had given birth two or three times were 81.8%, 14.9%, 2.7%, and 0.5%, respectively. The annual mean household income was 56,000 (SD: 25,600) USD, and the mean personal income was 19,300 (SD: 19,600) USD. Regarding employment status, 37.6% of the participants were homemakers, 48.6% were full-time workers, and 13.8% were part-time workers. The types of hospitals were as follows: one general hospital (n = 165, 29.7%), two obstetrics and gynecology hospitals (n = 161, 29.0%), and one university hospital (n = 230, 41.3%).

### Exploratory factor analysis

Half (n = 278, 50.0%) of the participants were randomly chosen. The KMO value was 0.931, and the *χ*^2^ value by Bartlett’s test of sphericity was 3596.483 (F = 231, p < 0.000), indicating a reasonable value for the factor analysis. The screen test showed that the number of factors was recommended to be between one and five. For the EFA, the multiple imputation method was used to handle missing data. The following variables and the rate of missing data for the individual IDDL items are shown in Table 1. The following three items for which the communality was less than 0.4 were excluded from the EFA: item 3 = 0.283, item 18 = 0.223, and item 22 = 0.053. The results of the EFA are shown in Table 2. The rate of women who had an EPDS total score of 13 or more was 9.2% (n = 51). The rate of women having a history of MDD based on the IDDL was 166 (29.9%). The medians and interquartile ranges of the EPDS scores were as follows: anxiety (2, 0–3), depression (0, 0–1), anhedonia (0, 0–0), total score (4, 1–8).

**Table 1:** Scores on the Japanese version of the IDDL.

	Multiply-imputed data set		Complete case data set			Multiply-imputed data set	Complete case data set	
	Mean	SD	Mean	SD	Missing (n)	Number over 2 weeks	Number over 2 weeks	Missing (n)
IDDL1	2.51	1.25	2.51	1.25	0	267	264	24
IDDL2	2.41	1.2	2.41	1.20	0	250	243	25
IDDL3	0.78	0.9	0.78	0.90	1	148	147	20
IDDL4	1.02	1.32	1.02	1.31	2	156	155	15
IDDL5	1.73	1.25	1.73	1.25	0	228	227	15
IDDL6	1.97	1.25	1.97	1.25	0	232	230	18
IDDL7	2.00	1.53	2.00	1.53	1	233	227	21
IDDL8	1.81	1.34	1.81	1.34	0	238	237	13
IDDL9	1.91	1.44	1.91	1.44	0	237	236	13
IDDL10	1.16	1.34	1.16	1.34	0	173	172	7
IDDL11	1.81	1.18	1.81	1.18	0	199	197	16
IDDL12	1.27	1.23	1.27	1.23	1	192	191	10
IDDL13	1.31	1.23	1.31	1.23	0	186	185	14
IDDL14	0.85	1.01	0.85	1.01	1	183	182	7
IDDL15	0.39	0.92	0.39	0.92	0	176	176	7
IDDL16	0.25	0.75	0.25	0.75	1	162	162	4
IDDL17	1.34	1.11	1.34	1.11	2	191	189	17
IDDL18	0.62	1.18	0.62	1.14	26	172	161	32
IDDL19	1.91	1.13	1.91	1.13	0	233	231	16
IDDL20	1.84	1.37	1.84	1.37	0	259	257	13
IDDL21	1.42	1.30	1.42	1.30	0	214	213	13
IDDL22	0.64	1.06	0.64	1.06	0	186	185	5
Total	30.96	16.85	30.95	16.85	34			102

åIDDL: Inventory to Diagnose Depression, Lifetime version; SD: standard deviation.

**Table 2:** EFA of the Japanese version of the IDDL (n = 278, maximum-likelihood estimation with promax rotation).

		Communality	1-factor	2-factor		3-factor			4-factor				5-factor				
			F1	F1	F2	F1	F2	F3	F1	F2	F3	F4	F1	F2	F3	F4	F5
IDDL1	Low mood	0.64	**0.77**	0.27	**0.57**	**0.88**	–0.07	–0.03	**0.51**	–0.13	0.33	0.15	**0.70**	–0.13	0.06	–0.01	0.20
IDDL2	Decreased energy	0.66	**0.81**	0.40	**0.48**	**0.71**	0.14	<0.01	**0.43**	0.04	0.29	0.16	**0.58**	0.05	0.09	0.01	0.18
IDDL4	Retardation	0.46	**0.62**	**0.52**	0.14	–0.04	**0.64**	0.18	0.18	**0.51**	–0.04	0.11	0.07	**0.52**	0.13	0.11	–0.02
IDDL5	Decreased interest in usual activities	0.73	**0.81**	**0.60**	0.27	**0.46**	0.42	–0.02	0.04	0.11	**0.74**	0.01	0.17	0.12	0.06	0.05	**0.58**
IDDL6	Decreased pleasure in usual activities	0.90	**0.84**	**0.54**	0.36	**0.62**	0.28	–0.05	0.04	–0.05	**1.00**	–0.09	0.19	–0.06	–0.03	0.05	**0.85**
IDDL7	Decreased libido	0.50	**0.55**	**0.83**	–0.24	0.04	**0.68**	–0.20	–0.42	0.33	**0.65**	0.06	–0.19	0.37	0.09	–0.14	**0.50**
IDDL8	Guilt	0.63	**0.78**	0.39	**0.45**	**0.48**	0.31	0.09	**0.39**	0.25	0.24	–0.01	**0.56**	0.31	–0.09	–0.08	0.09
IDDL9	Worthlessness	0.69	**0.79**	0.25	**0.61**	**0.51**	0.24	0.18	**0.51**	0.26	0.25	–0.14	**0.65**	0.32	–0.23	–0.06	0.09
IDDL10	Suicidal/death thoughts	0.61	**0.69**	0.12	**0.64**	**0.62**	0.02	0.17	**0.76**	0.04	–0.19	0.21	**0.86**	0.06	0.06	0.02	–0.24
IDDL11	Decreased concentration	0.66	**0.73**	**0.61**	0.17	–0.06	**0.79**	0.21	0.14	**0.64**	0.11	0.01	0.03	**0.66**	0.04	0.11	0.10
IDDL12	Indecisiveness	0.74	**0.70**	**0.65**	0.10	–0.15	**0.86**	0.18	0.11	**0.84**	–0.03	–0.03	0.05	**0.89**	–0.05	–0.03	–0.05
IDDL13	Decreased appetite	0.76	**0.61**	**1.02**	–0.37	0.07	**0.73**	–0.23	–0.24	0.28	0.01	**0.77**	–0.11	0.24	**0.74**	0.02	0.04
IDDL14	Weight loss	0.70	**0.56**	**0.87**	–0.27	0.33	**0.44**	–0.32	–0.11	–0.07	–0.04	**0.98**	0.16	–0.11	**0.83**	–0.03	–0.01
IDDL15	Increased appetite	0.54	**0.33**	–0.29	**0.66**	0.02	0.01	**0.66**	**0.61**	0.13	–0.19	–0.18	–0.04	0.10	0.05	**0.73**	–0.06
IDDL16	Weight gain	0.69	**0.32**	–0.46	**0.83**	0.17	–0.18	**0.71**	**0.70**	–0.01	–0.04	–0.33	0.04	–0.06	–0.09	**0.80**	0.08
IDDL17	Insomnia	0.42	**0.61**	**0.62**	0.03	0.23	**0.48**	–0.08	0.11	0.28	0.05	**0.31**	0.27	**0.29**	0.23	–0.08	<0.01
IDDL19	Anxiety	0.63	**0.76**	0.26	**0.57**	**0.81**	–0.02	0.01	**0.56**	–0.09	0.21	0.18	**0.73**	–0.09	0.09	0.01	0.11
IDDL20	Hopelessness	0.65	**0.73**	0.13	**0.68**	**0.80**	–0.08	0.09	**0.70**	–0.07	0.06	0.13	**0.89**	–0.05	<0.01	–0.03	–0.05
IDDL21	Irritability	0.40	**0.60**	0.12	**0.53**	**0.41**	0.11	0.22	**0.55**	0.11	–0.03	0.06	**0.46**	0.11	0.06	0.17	–0.04
Factor loading													48.64	9.85	5.36	4.61	3.93

EEA: exploratory factor analysis.

### Confirmatory factor analysis (CFA)

**Fig 1:**
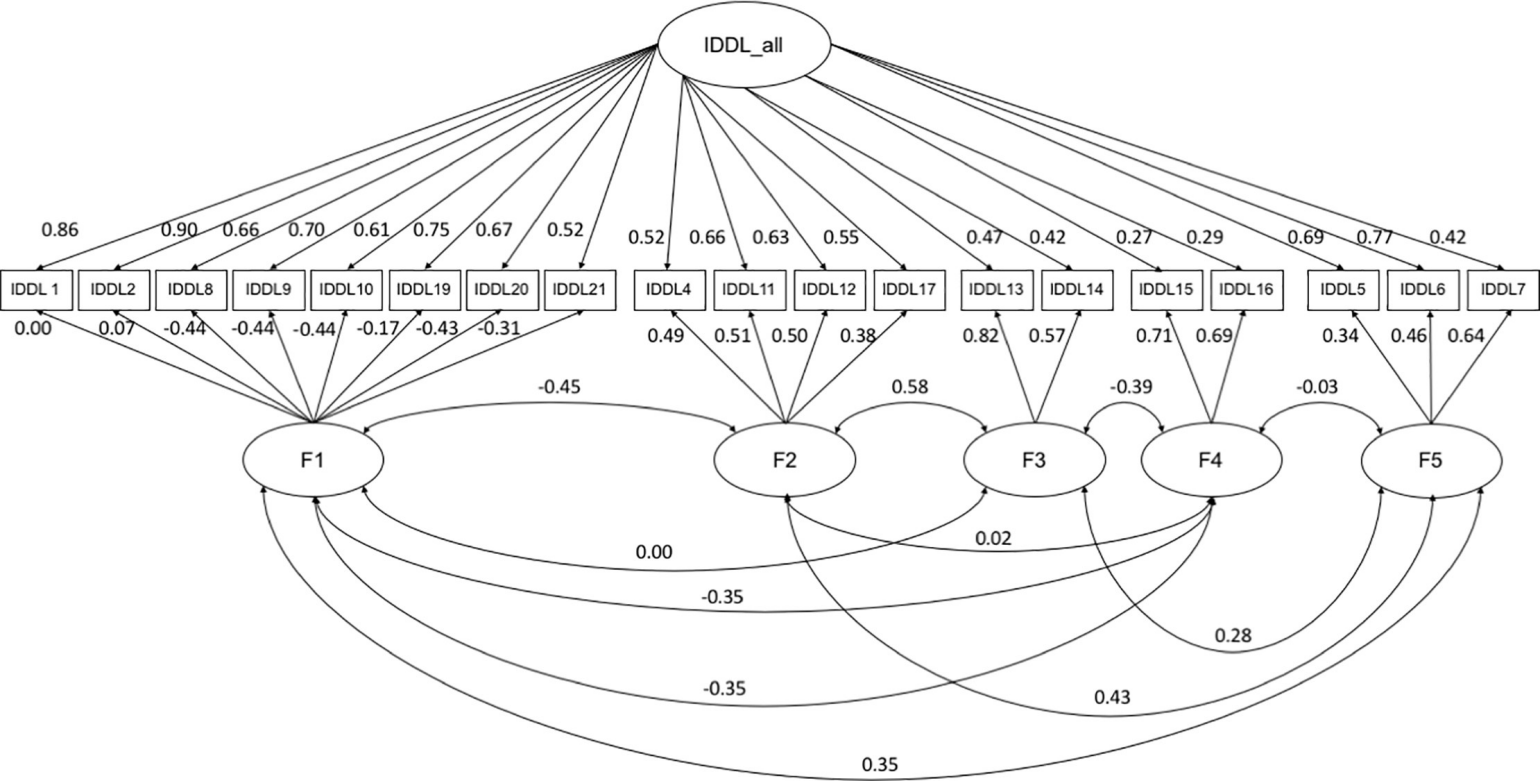
Best-fit model of the confirmatory factor analysis.

The other sample set (n = 276, 50.0%) was then used. For the CFA, full information maximum-likelihood estimation was used to handle missing data. Each of the factor structure models obtained by the EFA was examined. The results of the model fit by the CFA are shown in Table 3. The critical values of the *χ*^2^ distribution at the significance level of p < 0.001 corresponding to *df* values of 1, 2, 3, and 4 were 10.83, 13.82, 16.27, and 18.47, respectively. As shown in Table 3, Since these critical values were all lower than the Δ*χ*^2^/*df* values corresponding to the individual factor models, goodness of fit was shown to be improved by increasing the number of factors. The best-fit model of the CFA was the bifactor model, which consisted of a single general dimension along with the following five factors: (1) depression, anxiety, and irritability (items 1, 2, 8–10, and 19–21); (2) retardation, decreased concentration, indecisiveness, and insomnia (items 4, 11, 12, and 17); (3) decrease in appetite/significant weight loss (items 13 and 14); (4) increase in appetite/significant weight gain (items 15 and 16); and (5) diminished interest, pleasure, and libido (items 5–7). Cronbach’s α coefficients for these five factors were as follows: 0.910, 0.815, 0.780, 0.683, and 0.803, respectively. The correlations between these five factors are also shown in Fig 1. Each correlation was significant except for that between factors 3 and 4.

**Table 3:** CFA of the Japanese version of the IDDL.

Model	Items	χ^2^	df	χ^2^/df	p	Δχ^2^(df)	CFI	RMSEA	AIC
1-factor model	F1: 1, 2, 4–17, 19–21	848.36	152	5.58	p<0.000	-	0.784	0.129	962.36
2-factor model	F1: 4–7, 11–14, 17	716.21	151	4.74	p<0.000	132.15(1)	0.825	0.116	832.21
	F2: 1, 2, 8–10, 15, 16, 19–21								
3-factor model	F1: 1, 2, 5, 6, 8–10, 19–21	597.07	149	4.00	p<0.000	119.14(2)	0.861	0.104	707.07
	F2: 4, 7, 11–14, 17								
	F3: 15, 16								
4-factor model	F1: 1, 2, 8–10, 15, 16, 19–21	503.23	146	3.45	p<0.000	93.84(3)	0.889	0.094	629.23
	F2: 4, 11, 12								
	F3: 5, 6, 7								
	F4: 13, 14, 17								
5-factor model	F1: 1, 2, 8–10, 19–21	361.52	142	2.55	p<0.000	141.71(4)	0.932	0.075	495.52
	F2: 4, 11, 12, 17								
	F3: 13, 14								
	F4: 15, 16								
	F5: 5, 6, 7								
Bi-factor model*	F1: 1, 2, 8–10, 19–21	258.23	123	2.10	p<0.000	103.29(19)	0.958	0.063	430.23
	F2: 4, 11, 12, 17								
	F3: 13, 14								
	F4: 15, 16								
	F5: 5, 6, 7								
	F1-5: 1,2, 4–17, 19–21								

CFA: confirmatory factor analysis; CFI: comparative fit index; RMSEA: root mean square error of approximation; AIC: Akaike’s information criterion.

## Discussion

In the present study, we demonstrated the reliability and construct validity of the Japanese version of the IDDL for 556 pregnant women and revealed its factor structure. As a result, first, a bifactor model composed of a single general dimension along with the following five factors was extracted: (1) depression, anxiety, and irritability (items 1, 2, 8–10, and 19–21); (2) retardation, decreased concentration, indecisiveness, and insomnia (items 4, 11, 12, and 17); (3) decrease in appetite/significant weight loss (items 13 and 14); (4) increase in appetite/significant weight gain (items 15 and 16); and (5) diminished interest, pleasure, and libido (items 5–7). Second, the construct validity was confirmed. Third, according to the IDDL, the prevalence of pregnant women with a history of MDD was estimated to be 29.9%.

The original English and the Japanese version of the IDDL were validated for a small sample size [5, 6]. In the present study, reliability based on Cronbach’s α coefficient was shown to be sufficiently high using a large sample size of 556 pregnant women. However, since no clinical diagnostic interviews were conducted, its sensitivity and specificity could not be evaluated.

The present five factor structure was shown to have a sufficiently high Cronbach’s α value. However, higher values, such as those over 0.90, may reflect unnecessary duplication of content across items and point more to redundancy than to homogeneity [20]. Further research needs to consider the short version of the IDDL with similar items reduced.

As shown in Table 1, the items for which the factor loading value for a single general dimension was larger than that for five factors were all eight F1 items (depression, anxiety, and irritability), all four F2 items (retardation, decreased concentration, indecisiveness, and insomnia), and two F5 items (diminished interest and pleasure). By contrast, the items for which the factor loading value for a single general dimension was smaller than that for five factors were: all two F3 items (increase in appetite/significant weight gain), all two F4 items (increase in appetite/significant weight gain), and one F5 item (libido). These results may provide new evidence that mental symptoms are important factors contributing to depression, and that appetite and libido are physical symptoms secondary to mental symptoms.

The factor loading values of the F4 items (increase in appetite/significant weight gain) for a single general dimension were lower than those of the other 19 items, as shown in Fig 1. This finding was consistent with a previous study reporting that the prevalence of increased appetite was lower than that of decreased appetite in patients with MDD [21]; however, changes in appetite and weight are difficult to compare in MDD because the situation often varies depending on one’s physical health (e.g., obese individuals with physical disorders such as diabetes and comorbid diseases such as anorexia nervosa are at high risk for MDD).

Regarding the ratio of participants with a history of MDD using the IDDL, Zimmerman et al. [5] reported a prevalence of 15% among normal control probands, Uehara [22] reported a prevalence of 15% (14 of 93) among female Japanese workers, and Sakado [23] reported a prevalence of 12.7% (16 of 126) among employed Japanese adults; however, 29.9% of the pregnant women in the present study had a history of MDD, which is at least twice as high as previous reports. One of the reasons for the relatively high prevalence observed in our study may be a selection bias: 1) pregnant women who were in good mental health may not have had any interest in this study, and therefore, could have decided not to participate; and 2) in total, 41.3% of the participants were recruited from university hospitals, where large numbers of pregnant women who suffer from severe mental or physical comorbid illnesses are usually introduced from community hospitals.

The IDDL may estimate the rate of the prenatal women having a history of MDD significantly higher than the actual value; however, the false-positive rate could not be evaluated because no clinical interviews were conducted in this study. Clinical interviews should be performed to diagnose MDD in a future study.

Participants who were suspected of having a history of MDD based on the IDDL had significantly higher total EPDS scores than those who were not. The results of this study support previous findings that a history of MDD increases vulnerability to stress; therefore, confirming one’s history of MDD was useful for extracting those at high risk for perinatal depression. However, the IDDL could not confirm at what point the person concerned developed depression or experienced multiple depressive episodes. Therefore, it is considered preferable to make inquiries to the person concerned and confirm the times and instances of depressive episodes when evaluating the history of MDD in maternal health examinations using the IDDL.

This study did have a notable limitation. The sample size of 556 may have been insufficient to perform factor analyses. Further research on factor analyses using larger samples is needed to identify a more robust factor structure than that observed in the present study. A concurrent validity study using clinical diagnoses by the DSM-5 and cross-cultural research may also be needed.

In conclusion, the internal reliability, construct validity, and factor structure of the Japanese version of the IDDL for pregnant women was demonstrated. A bifactor model composed of a single general dimension along with the following five factors was extracted: (1) depression, anxiety, and irritability; (2) retardation, decreased concentration, indecisiveness and insomnia; (3) decrease in appetite/significant weight loss; (4) increase in appetite/significant weight gain; and (5) diminished interest, pleasure, and libido.

## Supporting information

S1 Dataset (XLSX)
